# Conditioned medium from bone marrow-derived mesenchymal stem cells inhibits vascular calcification through blockade of the BMP2–Smad1/5/8 signaling pathway

**DOI:** 10.1186/s13287-018-0894-1

**Published:** 2018-06-13

**Authors:** Shuangshuang Wang, Siwang Hu, Jian Wang, Yahui Liu, Ruochi Zhao, Maoqing Tong, Hanbin Cui, Nan Wu, Xiaomin Chen

**Affiliations:** 1 Department of Cardiology, Ningbo First Hospital, Ningbo, 315000 China; 2 Spine Tumor Center, Changzheng Hospital, Second Military Medical University, Shanghai, 200003 China

**Keywords:** Vascular calcification, Conditioned medium from bone marrow-derived mesenchymal stem cells, BMP2–Smad1/5/8 signaling, Atherosclerosis

## Abstract

**Background:**

Arterial calcification is associated with cardiovascular disease as a complication of advanced atherosclerosis and is a significant contributor to cardiovascular morbidity and mortality. Osteoblastic differentiation of vascular smooth muscle cells (VSMCs) plays an important role in arterial calcification and is characterized by cellular necrosis, inflammation, and lipoprotein and phospholipid complexes, especially in atherosclerotic calcification. The conditioned medium from bone marrow-derived mesenchymal stem cells (MSC-CM) is well known as a rich source of autologous cytokines and is universally used for tissue regeneration in current clinical medicine. Here, we demonstrate that MSC-CM inhibits beta-glycerophosphate (β-GP)-induced vascular calcification through blockade of the bone morphogenetic protein-2 (BMP2)–Smad1/5/8 signaling pathway.

**Methods:**

VSMC calcification was induced by β-GP followed by treatment with MSC-CM. Mineral deposition was assessed by Alizarin Red S staining. Intracellular calcium content was determined colorimetrically by the *o*-cresolphthalein complexone method and alkaline phosphatase (ALP) activity was measured by the *para*-nitrophenyl phosphate method. Expression of BMP2, BMPR1A, BMPR1B, BMPR2, msh homeobox 2 (Msx2), Runt-related transcription factor 2 (Runx2), and osteocalcin (OC), representative osteoblastic markers, was assessed using real-time polymerase chain reaction analysis while the protein expression of BMP2, Runx2, and phosphorylated Smad1/5/8 was detected by western blot analysis.

**Results:**

Our data demonstrated that MSC-CM inhibits osteoblastic differentiation and mineralization of VSMCs as evidenced by decreased calcium content, ALP activity, and decreased expression of BMP-2, Runx2, Msx2, and OC. MSC-CM suppressed the expression of phosphorylated Smad1/5/8 and the β-GP-induced translocation from the cytoplasm to the nucleus. Further study demonstrated that human recombinant BMP-2 overcame the suppression of VSMC calcification by MSC-CM.

**Conclusion:**

MSC-CM may act as a novel therapy for VSMC calcification by mediating the BMP2–Smad1/5/8 signaling pathway

## Background

Vascular calcification is a common feature of coronary artery disease as a complication of advanced atherosclerosis and is highly associated with aging, chronic kidney disease, diabetes, hypertension, and dyslipidemia [1, 2]. Calcification is a common finding in human atherosclerotic lesions, and arterial calcification is generally classified into calcification within an atherosclerotic plaque. During atherosclerosis, calcification develops in the arterial intima. Whereas medial calcification is a hallmark of diabetes and chronic kidney disease, arterial calcification is clearly associated with the mortality risk in individuals with atherosclerosis and diabetes. It has been reported that a spotty pattern of calcification is associated with coronary unstable ruptured plaques in patients with acute myocardial infarction [3, 4].

Increasing evidence now supports that osteo/chondrocytic transformation of vascular smooth muscle cells (VSMCs) and their dedifferentiation from a contractile to a proliferative phenotype are important for the initiation and progression of vascular calcification [1–3]. It is not clear to what extent *trans*-differentiation of VSMCs contributes to the pathogenesis of vascular calcification in vivo, although many in-vitro studies have demonstrated that VSMCs can undergo osteogenic differentiation and calcification [5–7]. Unfortunately, there are currently no ideal therapies directed at the prevention or treatment of vascular calcification to combat this growing problem in clinical practice today.

Bone marrow-derived mesenchymal stem cells (MSCs) have received a lot of attention for their efficacy as a medical therapeutic strategy, and have been shown to be efficacious for a variety of diseases such as myocardial infarction, corneal wounds, and lung injury; their efficacy has, in part, been attributed to their anti-inflammatory properties [8–10]. The conditioned medium from MSCs (MSC-CM) is well known as a rich source of autologous cytokines and is universally used in current clinical medicine. Recent studies have provided compelling evidence that vascular calcification is associated with inflammatory status and is enhanced by inflammatory cytokines, especially in atherosclerosis and diabetes [11–13]. MSC-CM is known to have potential anti-inflammatory activity. However, the functional role of MSC-CM in vascular calcification has not yet been elucidated. In this article, we investigated the effect of MSC-CM on VSMC calcification and the underlying mechanism.

## Methods

### Reagents, antibodies, and assay kits

Mouse MSCs (C57BL/6 mouse background) were purchased from Cyagen Biosciences (Guangzhou, China) and mouse VSMCs (C57BL/6 mouse background) were purchased from Procell Life Science & Technology (Wuhan, China). Beta-glycerophosphate (β-GP, G9422) and Alizarin Red S (A5533) were obtained from Sigma. Antibodies for BMP-2 (ab6285), Runx2 (ab76956), and p-Smad1/5/8 (ab66737) were purchased from Abcam. rhBMP-2 (B3555) and Noggin were purchased from Cyagen Biosciences. The Calcium Colorimetric Assay was obtained from Biovision, USA, and LabAssay ALP was purchased from Wako Pure Chemical Industries, Japan. The BCA protein assay was obtained from Pierce, USA.

### Cell culture

MSCs and VSMCs were routinely cultured in complete medium containing Dulbecco’s Modified Eagle’s Medium (DMEM) with 4500 mg/L d-glucose, 4.00 nM l-glutamine, and sodium pyruvate supplemented with 10% FBS, 100 U/ml of penicillin and 100 μg/ml of streptomycin. The cells were incubated at 37 °C in a humidified atmosphere with 5% CO_2_ and passaged at confluence with 0.5% trypsin/EDTA. VSMCs between passages 8 and 11 were used in this study.

The MSC-CM was collected every other day, and the suspended cells and debris were removed by centrifuging at 800×*g* for 10 min and filtering through a 50-μm mesh and stored at − 80 °C until further use.

VSMCs were treated with DMEM medium (NC group), DMEM medium containing 10 mM β-GP (DMEM^β-GP^ group), or MSC-CM with 10 mM β-GP (MSC-CM^β-GP^ group). The medium was changed every 2 days.

### Alizarin Red staining and quantification

Mineral deposition in cultured VSMCs was assessed by Alizarin Red staining. Calcified VSMCs were fixed in 95% alcohol for 15 min and exposed to 2% Alizarin Red (pH 4.2) for 5 min at room temperature. Cells were washed with deionized water to remove excess dye and images were taken using an inverted microscope. For Alizarin Red staining quantification, 10% formic acid was used to elute Alizarin Red dye and the absorbance at 405 nm was determined with a microplate reader. Other plates of VSMCs treated with the same condition were used to normalize to protein content.

### Intracellular calcium content

For quantification of calcium content, cells were washed with PBS and decalcified with 0.6 mM HCl at 4 °C for 24 h. Calcium released from the cell cultures into the supernatant was determined colorimetrically by the *o*-cresolphthalein complexone method using the Calcium Colorimetric Assay and normalized to protein content. The protein content was determined using BCA protein assay (Pierce, USA).

### Alkaline phosphatase activity assay

ALP activity of VSMCs was measured using LabAssay ALP (Wako Pure Chemical Industries), according to the manufacturer’s protocol. ALP activity was normalized to total protein determined by BCA protein assay.

### Reverse transcription PCR and real-time quantitative PCR

To examine whether MSC-CM inhibits osteoblastic phenotype formation, the expression of BMP2, BMPR1A, BMPR1B, BMPR2, Msx2, Runx-2, and osteocalcin, representative osteoblastic markers, was assessed using real time-polymerase chain reaction analysis. Total RNA was isolated from cultured VSMCs using TRIzol Reagent (Invitrogen Corp, Carlsbad, CA, USA) according to the manufacturer’s instructions. Reverse transcription was performed using an RT-PCR kit (Takara Biotech). Levels of mRNA were quantitated using an ABI 7500HT Fast Real-Time PCR System (Applied Biosystems, Grand Island, NY, USA). Glyceraldehyde-3-phosphate dehydrogenase (GAPDH) was used as an endogenous control. The 2^–ΔΔCt^ method was used to calculate relative expression levels. Sequence-specific primers for the genes were designed using Premier 5 software and are presented in Table 1.

**Table 1:** Sequence-specific primers

Gene		Sequence (5′–3′)
*BMP2*	Forward	TCTTCCGGGAACAGATACAGG
	Reverse	TGGTGTCCAATAGTCTGGTCA
*BMPR1A*	Forward	GGCCATTGCTTTGCCATTATAG
	Reverse	CTTTCGGTGAATCCTTGCATTG
*BMPR1B*	Forward	CCGACCTCGGTACAGCATTG
	Reverse	GCTCTGAGACTGCTCGATCAAG
*BMPR2*	Forward	TTGGGATAGGTGAGAGTCGAAT
	Reverse	TGTTTCACAAGATTGATGTCCCC
*Msx2*	Forward	GGAGCACCGTGGATACAGG
	Reverse	TAGAAGCTGGGATGTGGTGAA
*Runx2*	Forward	ATGCTTCATTCGCCTCACAAA
	Reverse	GCACTCACTGACTCGGTTGG
*osteocalcin*	Forward	TGTCTTCTCCACAGCCTTCATG
	Reverse	ACCACTCCAGCACAACTCCTTC
*GAPDH*	Forward	ACCACAGTCCATGCCATCAC
	Reverse	TCCACCACCCTGTTGCTGTA

### Western blotting

Cells were harvested and centrifuged at 1000×*g* at 4 °C for 5 min, after which the supernatant was discarded and the samples were prepared using lysis buffer with protease inhibitors (1 mmol/L benzamidine, 1 μg/ml leupeptin, 10 μg/ml soybean trypsin inhibitor, and 0.5 mmol/L PMSF). Samples were centrifuged at 14,000×*g* for 5 min and the supernatant was collected. The total protein content of the samples was measured using the BCA method, and sample aliquots containing equal amounts of protein were boiled for 5 min in sample loading buffer and separated by sodium dodecylsulphate polyacrylamide gel electrophoresis (SDS-PAGE) followed by PVDF membrane transfer (Immobilon-P; Millipore). Membranes were subsequently blocked with 5% nonfat dry milk in TBS containing 0.1% Tween 20 (TBST). Next, PVDF membranes were incubated with antibodies for BMP-2, Runx2, p-Smad1/5/8, or anti-β-actin (1:3000 diluted in TBS-T) overnight at 4 °C, followed by an appropriate secondary antibody (horseradish peroxidase-conjugated anti-goat IgG) for 1 h at room temperature. The reaction was visualized by enhanced chemiluminescence (ECL)/enhanced chemiluminescent system (Amersham Biosciences). Band intensities were quantified by ImageJ software (Wayne Rasband, NIH)/a VersaDoc (BioRad) scanning system and densitometry values were normalized to that of β-actin.

### Statistical analysis

Data were expressed as mean ± standard deviation of three independent experiments. Comparisons of parameters among groups were made by one-way ANOVA, followed by a homogeneity of variance test. Differences were considered statistically significant when *P* < 0.05.

## Results

### β-GP induced calcium deposition in VSMCs in a dose-dependent manner

**Fig. 1:**
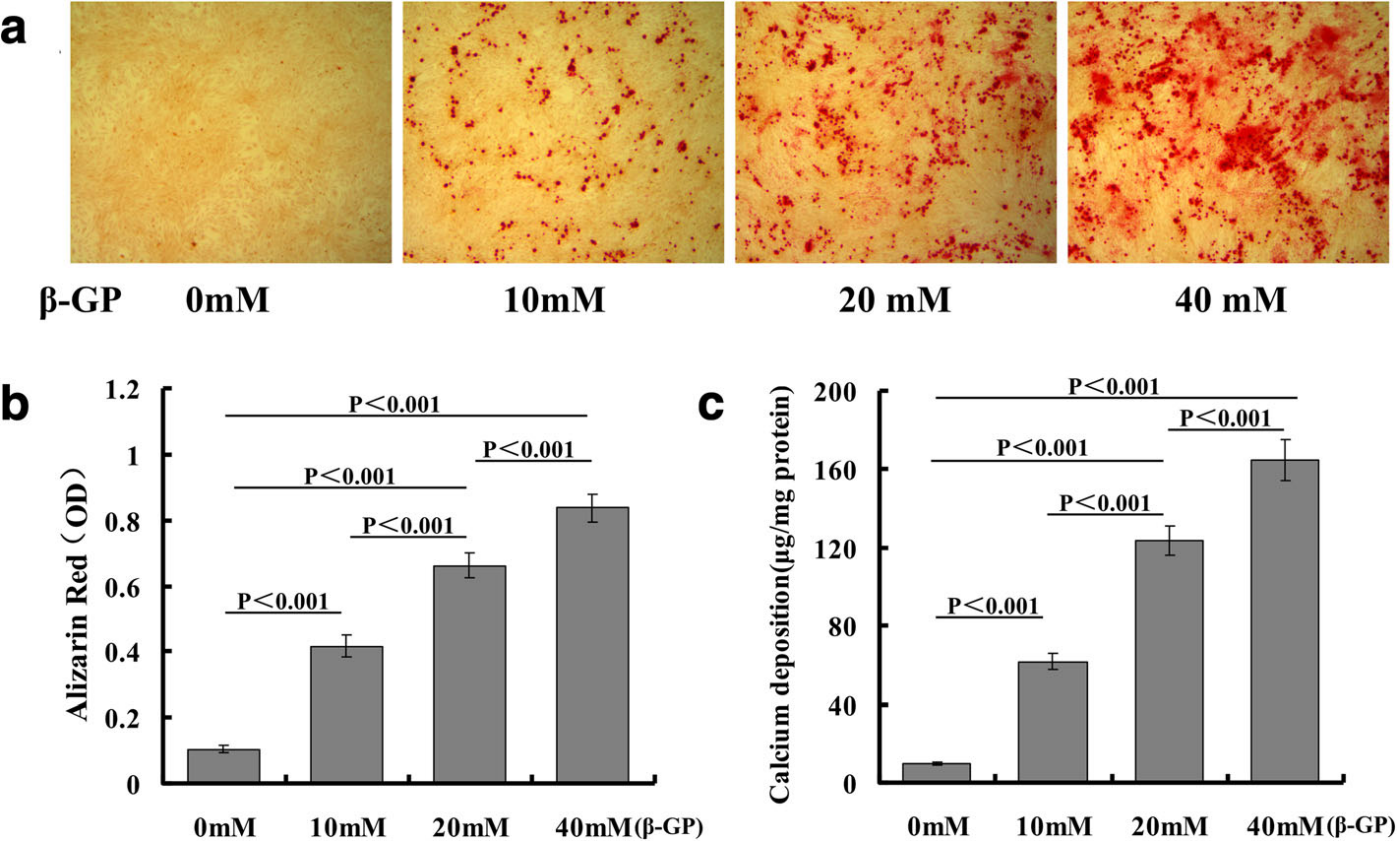
β-GP induced calcium deposition in VSMCs in a dose-dependent manner. VSMCs cultured in normal medium or in calcification medium with 10, 20 and 40 mmol/l of β-GP for 14 days. **a** Alizarin Red staining of microscopic views (×100). **b** Formic acid 10% used to elute Alizarin Red dye and quantification of Alizarin Red staining measured with a microplate reader and normalized to total protein content. **c** Calcium content shown as mean ± standard deviation of three independent experiments conducted in duplicate. β-GP beta-glycerophosphate, OD optical density

The VSMC calcification model was established by culture in the medium supplemented with 10, 20, or 40 mM β-GP. The results of the Alizarin Red staining and quantification showed that β-GP induced a dose-dependent increase in calcium deposition in VSMCs (Fig. 1a, b). Further, the calcium content showed a dose-dependent increase (Fig. 1c).

### MSC-CM inhibits β-GP-induced calcium deposition in VSMCs

**Fig. 2:**
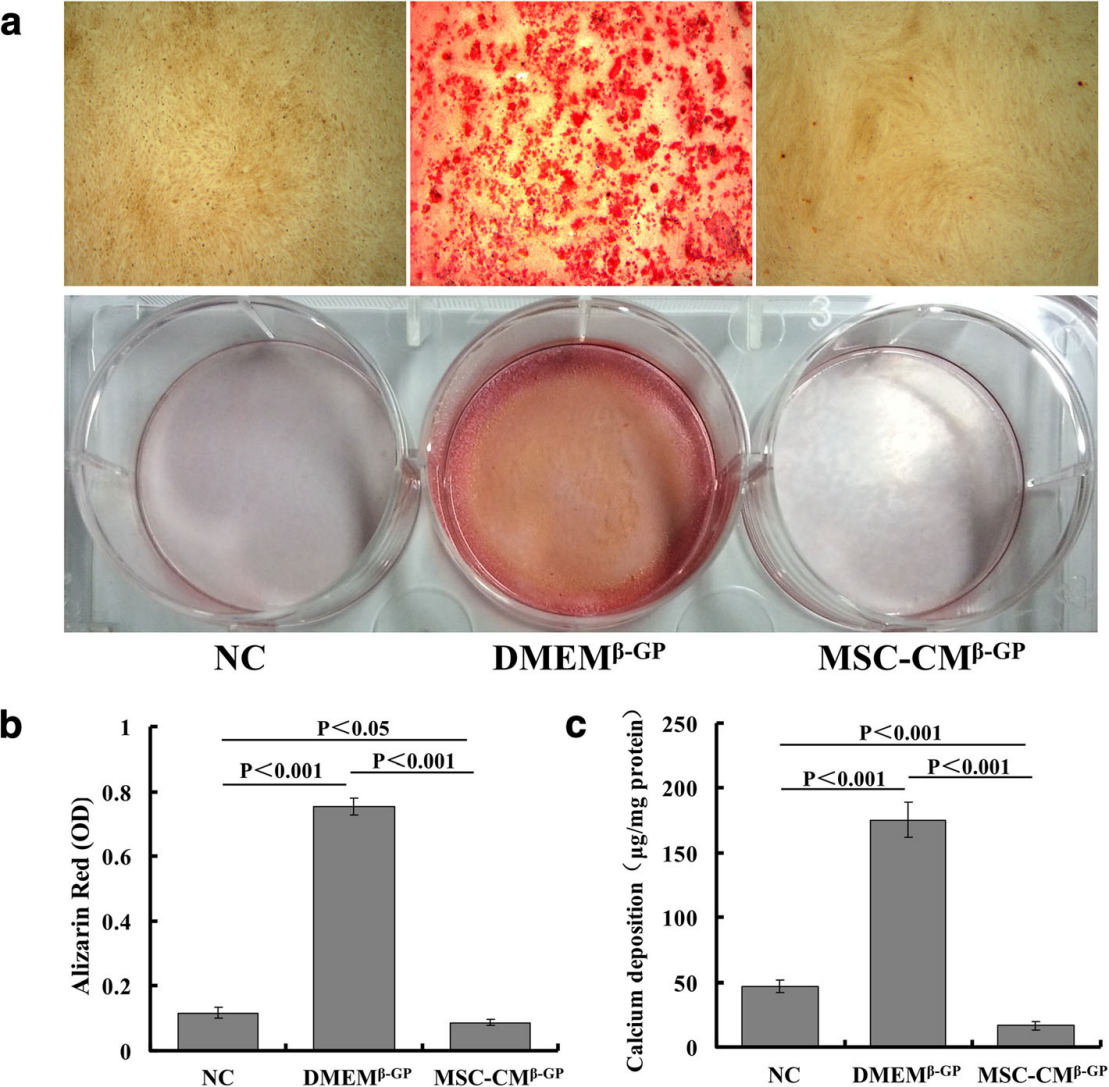
MSC-CM inhibits β-GP-induced calcium deposition in VSMCs. Confluent cells incubated with normal medium or calcification medium with or without MSC-CM for 14 days. Calcification induced by addition of β-GP (10 mM). **a** Representative Alizarin Red staining of microscopic views (×100, upper) and plates (lower). **b** Quantification of Alizarin Red staining. **c** Calcium contents measured and normalized by protein content of cell lysates. β-GP beta-glycerophosphate, DMEM Dulbecco’s Modified Eagle’s Medium, DMEM^β-GP^ DMEM medium containing 10 mM β-GP, MSC-CM conditioned medium from bone marrow-derived mesenchymal stem cells, MSC-CM^β-GP^ MSC-CM with 10 mM β-GP, NC DMEM medium, OD optical density

To determine the effect of the MSC-CM on VSMC calcification, we investigated the calcium deposition and calcium content in VSMCs. Compared with the NC group, 10 mM β-GP induced a significant increased calcium deposition of VSMCs (DMEM^β-GP^ group), as determined by Alizarin Red staining and quantification. In contrast, MSC-CM almost totally suppressed β-GP-induced calcium deposition in VSMCs (MSC-CM^β-GP^ group) (Fig. 2a, b).

Next, to further confirm the inhibitory effect of MSC-CM on calcification, we performed intracellular calcium content analysis in VSMCs. As shown in Fig. 2c, the calcium content was highly elevated in the medium with β-GP compared with the normal medium (DMEM^β-GP^ group vs NC group). In contrast, the calcium content was decreased obviously in the MSC-CM^β-GP^ group, compared to the DMEM^β-GP^ group.

Taken together, these data indicate that MSC-CM treatment attenuated the osteoblastic differentiation and calcification of VSMCs. These results indicate that MSC-CM may be a novel therapeutic agent for VSMC calcification.

### MSC-CM abolished β-GP-induced ALP activity and expression of specific-osteogenic markers in VSMCs

**Fig. 3:**
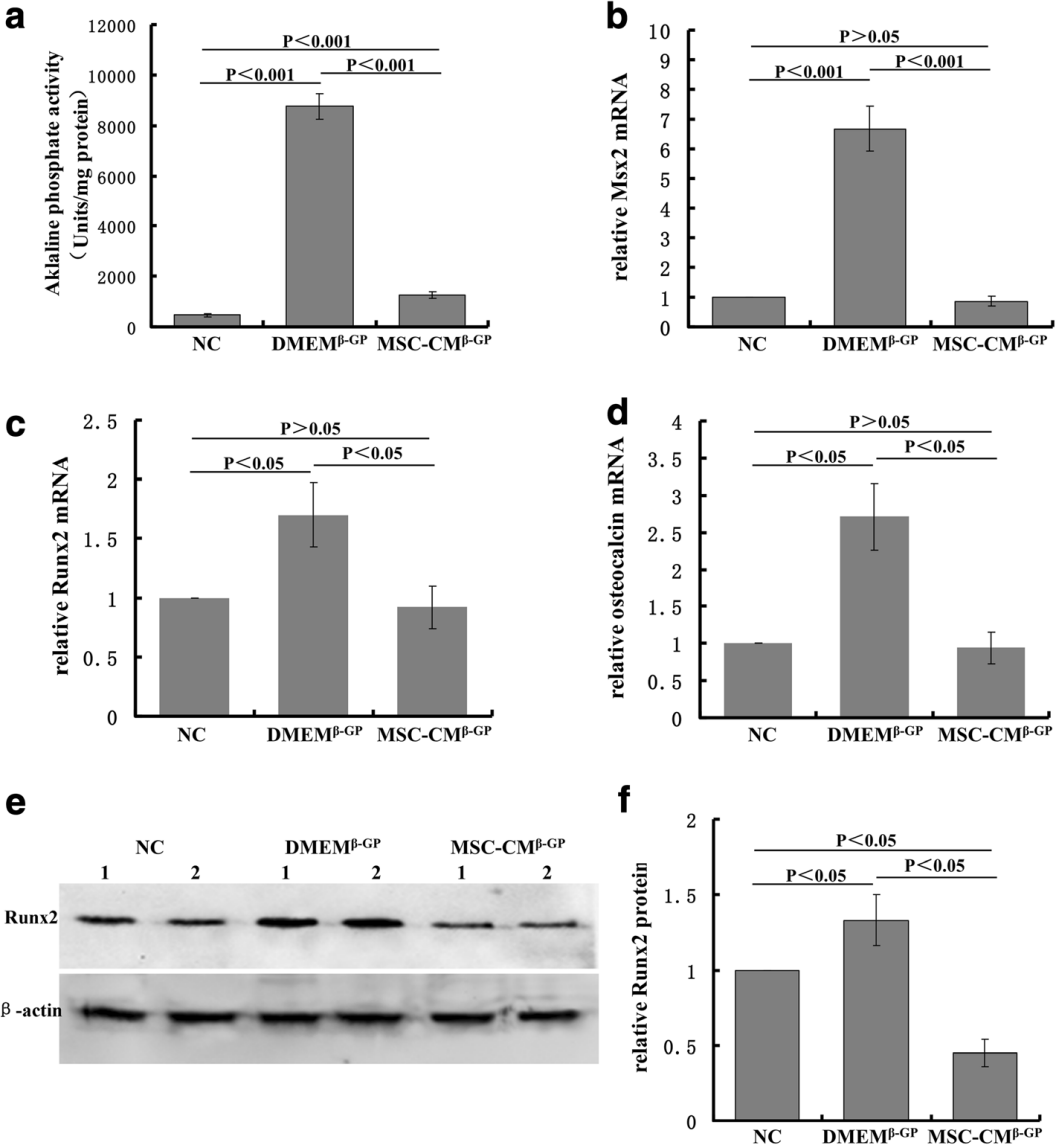
MSC-CM abolished β-GP-induced ALP activity and expression of specific osteogenic markers in VSMCs. Confluent VSMCs incubated with different media in different groups. **a** ALP activity measured after 3 days by ALP kit, normalized to cellular protein contents. Levels of Msx2 (**b**), Runx2 (**c**) and Osteocalcin (**d**) mRNA determined after 7 days by rt-PCR. **e** Representative immunoblot of Runx2. **f** Runx2 protein quantified by densitometry. β-GP beta-glycerophosphate, DMEM Dulbecco’s Modified Eagle’s Medium, DMEM^β-GP^ DMEM medium containing 10 mM β-GP, MSC-CM conditioned medium from bone marrow-derived mesenchymal stem cells, MSC-CM^β-GP^ MSC-CM with 10 mM β-GP, Msx2 msh homeobox 2, NC DMEM medium, Runx2 Runt-related transcription factor 2

To further investigate the inhibitory effect of MSC-CM on VSMC calcification, we detected ALP activity and the expression of specific-osteogenic markers such as Runx2, Msx2, and osteocalcin in different VSMC groups. First, ALP activity was highly increased in the medium with β-GP compared with normal medium and this increase was significantly reduced by MSC-CM (Fig. 3a). Next, we observed that the expressions of Runx2, Msx2, and osteocalcin mRNA were increased when VSMCs were treated with β-GP. However, the increased mRNA expression of these markers was abolished by MSC-CM treatment (Fig. 3b–d). Runx2 protein expression was also demonstrably induced by β-GP and suppressed by MSC-CM (Fig. 3e, f).

### MSC-CM blocks BMP2–Smad1/5/8 signaling pathway

**Fig. 4:**
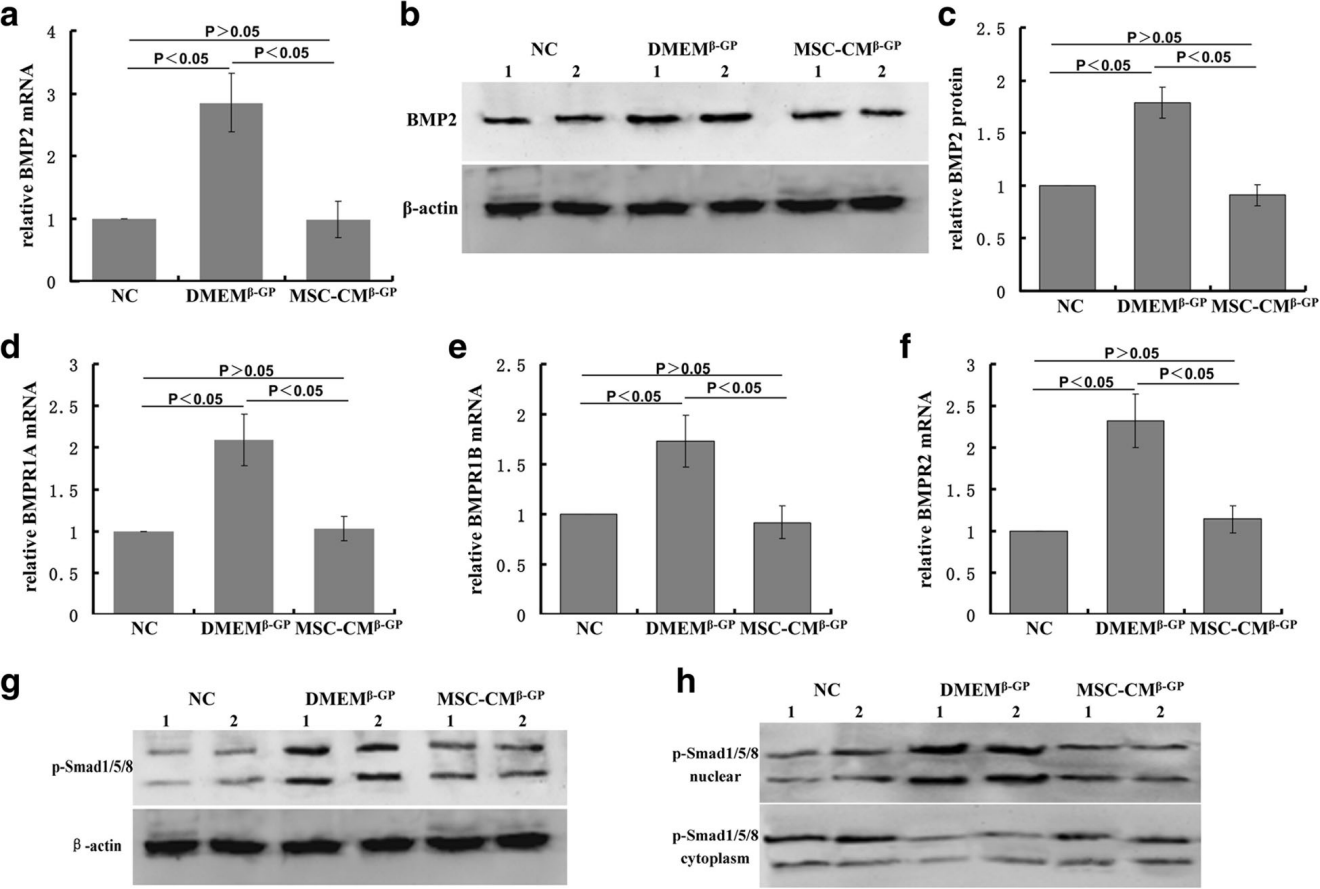
MSC-CM blocks BMP2–Smad1/5/8 signaling pathways. Confluent VSMCs incubated with different media in different groups for 7 days. **a** Effect of MSC-CM on BMP-2 mRNA expression. **b** Representative immunoblot of BMP2. **c** BMP2 protein quantified by densitometry. Levels of (**d**) BMPR1a, (**e**) BMPR1b, and (**f**) BMPR2 mRNA determined by rt-PCR. **g** Phosphorylation of Smad1/5/8 in whole cells. **h** Phosphorylation of Smad1/5/8 in nucleus and cytoplasm. β-GP beta-glycerophosphate, BMP-2 bone morphogenetic protein-2, DMEM Dulbecco’s Modified Eagle’s Medium, DMEM^β-GP^ DMEM medium containing 10 mM β-GP, MSC-CM conditioned medium from bone marrow-derived mesenchymal stem cells, MSC-CM^β-GP^ MSC-CM with 10 mM β-GP, NC DMEM medium

We discovered that MSC-CM inhibited the BMP2 expression of VSMCs. In this experiment, we evaluated the mRNA and protein expression of BMP2 in cells treated with β-GP and/or MSC-CM. First, the expression of BMP2 mRNA and protein were increased when VSMCs were treated with β-GP. However, the increased mRNA and protein expression of BMP2 was suppressed by MSC-CM (Fig. 4a–c).

BMP2–Smad signaling is related to the BMP receptors and Smad transcription factors in osteogenesis [14]. The induction of BMP2 by treatment with β-GP and the inhibition of BMP2 by treatment with MSC-CM in VSMCs prompted us to investigate whether or not MSC-CM can repress the BMP receptor–Smad signaling pathway. Next, we investigated the expressions of BMP receptors in our model. As shown in Fig. 4d–f, BMPR1a, BMPR1b, and BMPR2 mRNA levels were induced 3 days after β-GP treatment. MSC-CM, on the other hand, inhibited the expression of BMPR1a, BMPR1b, and BMPR2 mRNA levels.

After the activation of BMP receptors, the expression and translocation of phosphorylated Smad1/5/8 from the cytoplasm to the nucleus may trigger osteoblast differentiation. Therefore, we examined whether MSC-CM regulated phospho-Smad1/5/8 expression in whole cells and nuclear and cytoplasm fractions, separately. We found that phospho-Smad1/5/8 expression was increased after β-GP treatment in VSMCs. The increase in phospho-Smad1/5/8 protein expression was completely suppressed by treatment with MSC-CM (Fig. 4g, h). Phosphorylated Smad1/5/8 in control VSMCs is normally expressed minimally in the nucleus but higher in the cytoplasm. When VSMCs were stimulated by β-GP, however, phosphorylated Smad1/5/8 was mainly distributed in the nucleus, not in the cytoplasm. After treatment with MSC-CM, expression of phospho-Smad1/5/8 in the nucleus regressed while the cytoplasmic expression increased.

### BMP2 overcomes the inhibited effect of MSC-CM on β-GP-induced VSMC calcification

**Fig. 5:**
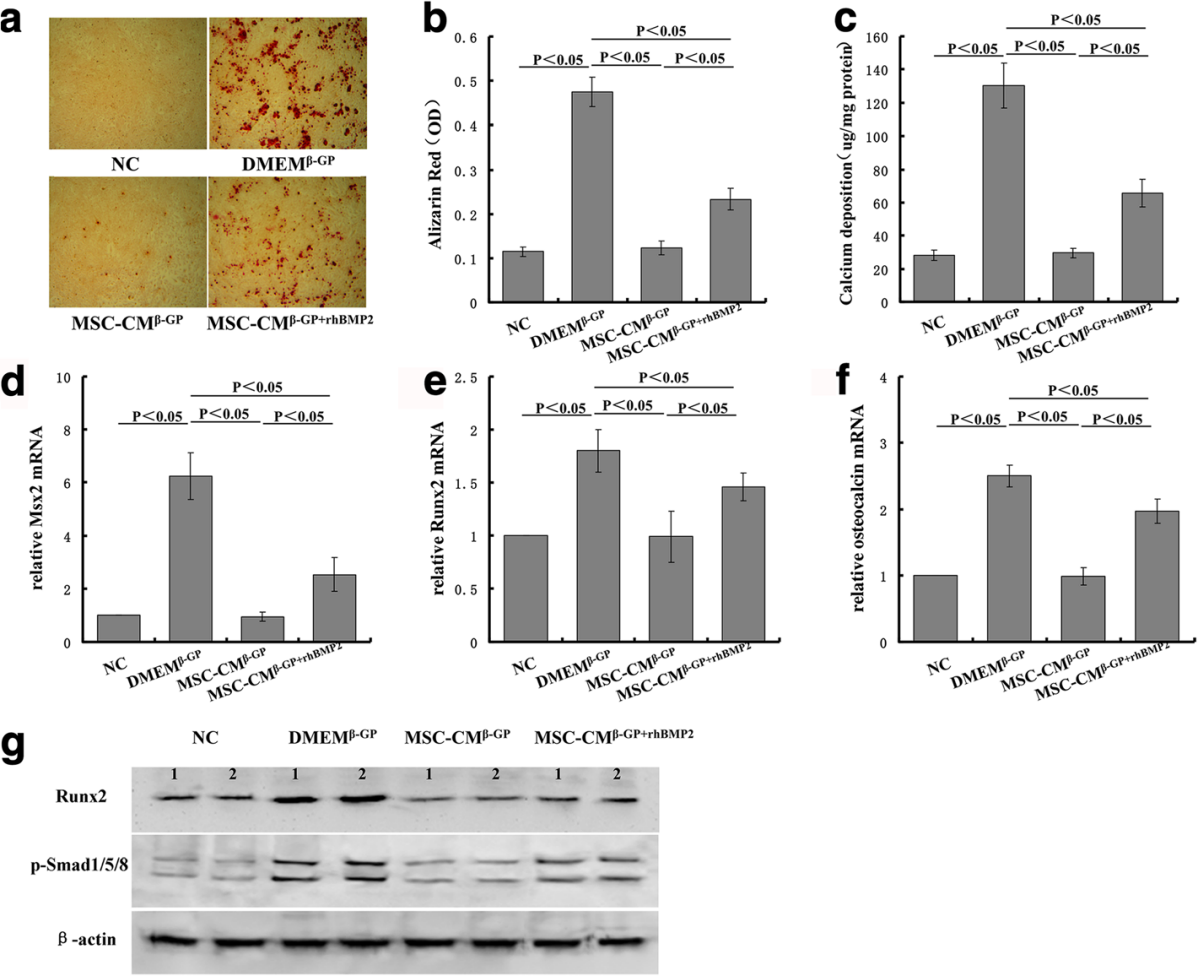
BMP2 overcomes inhibited effect of MSC-CM on β-GP-induced VSMC calcification. **a** Microscopic views (×100) of Alizarin Red staining. **b** Quantification of Alizarin Red staining. **c** Calcium contents measured and normalized by protein content of cell lysates. Expression of Msx2 (**d**), Runx2 (**e**), and osteocalcin (**f**) mRNA assessed by rt-PCR. **g** Cell lysates analyzed by western blot analysis with anti-Runx2 and p-Smad1/5/8. β-GP beta-glycerophosphate, DMEM Dulbecco’s Modified Eagle’s Medium, DMEM^β-GP^ DMEM medium containing 10 mM β-GP, MSC-CM conditioned medium from bone marrow-derived mesenchymal stem cells, MSC-CM^β-GP^ MSC-CM with 10 mM β-GP, Msx2 msh homeobox 2, NC DMEM medium, OD optical density, Runx2 Runt-related transcription factor 2

To determine whether human recombinant BMP2 (rhBMP2) can reverse the inhibitory effect of MSC-CM on β-GP-induced calcification deposition, VSMCs were incubated with MSC-CM containing 200 ng/ml of rhBMP2. The concentrations of BMP-2 used were chosen based on previous study [5] and the potency of commercial preparations as indicated by the manufacturers. MSC-CM significantly decreased the calcium deposition and calcium content in VSMCs, which was overcome by rhBMP2 (Fig. 5a–c). Next, we found that rhBMP2 upregulated the expression of Runx2, Msx2, and osteocalcin suppressed by MSC-CM (Fig. 5d–f). rhBMP2 also significantly increased Runx2, and phospho-Smad1/5/8 protein expression was inhibited by MSC-CM (Fig. 5g).

### Noggin, BMP2 antagonist, suppresses VSMC calcification by inhibiting Smad1/5/8 signaling

We examined whether calcification induced by β-GP was mediated by BMP2. To test this hypothesis, the BMP antagonist, Noggin, was added to VSMCs prior to the addition of β-GP. VSMCs were pretreated with vehicle or Noggin (100 ng/ml) for 2 h followed by incubation with β-GP. Calcification, calcium deposition, osteoblast differentiation markers, and BMP2–Smad signaling were then investigated.

**Fig. 6:**
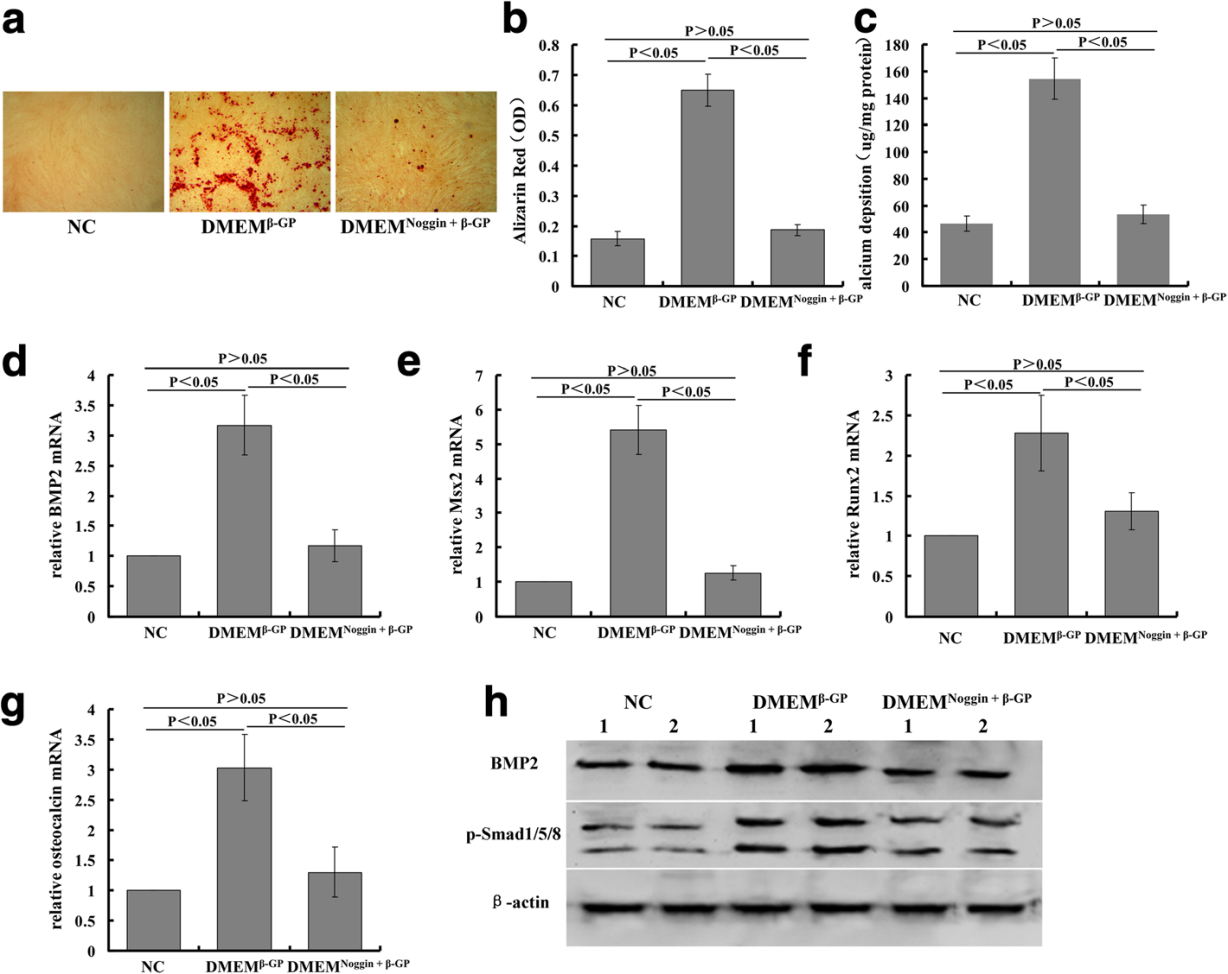
Noggin, BMP2 antagonist, suppresses VSMC calcification by inhibiting Smad1/5/8 signaling. VSMCs pretreated with Noggin (100 ng/ml) or vehicle for 2 h followed by incubation with 10 mM β-GP. **a** Alizarin Red staining (×100). **b** Quantification of Alizarin Red staining. **c** Calcium contents measured and normalized by protein content of cell lysates. mRNA expression of (**d**) BMP2, (**e**) Msx2, (**f**) Runx2, and (**g**) osteocalcin analyzed by rt-PCR. **h** Protein expression of BMP2 and phospho-Smad1/5/8 performed by western blot analysis. β-GP beta-glycerophosphate, DMEM Dulbecco’s Modified Eagle’s Medium, DMEM^β-GP^ DMEM medium containing 10 mM β-GP, DMEM^Noggin+β-GP^ DMEM medium containing 10 mM β-GP and 100 ng/ml Noggin, Msx2 msh homeobox 2, NC DMEM medium, OD optical density, Runx2 Runt-related transcription factor 2

Noggin suppressed β-GP-induced calcium deposition in VSMCs, as determined by Alizarin Red staining and quantification (Fig. 6a, b). In addition, the increased intracellular calcium content induced by β-GP was also inhibited by Noggin (Fig. 6c).

Quantitative real-time PCR demonstrated that Noggin blocked the β-GP-induced osteoblast differentiation markers BMP2, Runx2, Msx2, and osteocalcin in VSMCs (Fig. 6d–g). Western blot analysis similarly showed that Noggin decreases β-GP-induced BMP2 protein expression in VSMCs (Fig. 6h). Further, we investigated whether Noggin alters the expression of phospho-Smad1/5/8 protein in VSMCs. As predicted, the increased expression of phospho-Smad1/5/8 was blocked by treatment with Noggin (Fig. 6h).

## Discussion

Vascular calcification is highly prevalent in many kinds of diseases including aging, atherosclerosis, diabetes, hypertension, chronic kidney disease, and dyslipidemia. In coronary arteries, calcium deposits influence atherosclerotic plaque stability and increase the incidence of acute coronary syndrome, depending on the size and distribution of the deposits [15]. In addition, the deposition of calcium minerals in the vessels, especially the medial arteries, ultimately results in vascular stiffening, increased pulse wave velocity, and increased pulse pressure, which therefore increases cardiac work, and eventually leads to left ventricular hypertrophy and diastolic dysfunction [15]. Vascular calcification is thus associated with a 3-fold to 4-fold increased risk for cardiovascular morbidity and mortality [4]. It resembles skeletal osteogenesis, and many bone cells as well as bone-related factors involved in both formation and resorption have been localized in calcified arteries.

Bone marrow-derived mesenchymal stem cells (MSCs) have received a lot of attention for their efficacy as a medical therapeutic strategy and have been regarded as a future cell-based therapy. Recently, the therapeutic benefits of intravenous or local MSC transplantation have been observed in many diseases and injury models including acute lung injury [10, 16], myocardial infarction [17], acute renal failure [18], cerebral ischemia [19], Alzheimer’s disease [20], and corneal damage [21]. However, low survival rates and the potential tumorigenicity of implanted MSCs could undermine the efficacy of cell-based therapy. The conditioned medium from MSCs (MSC-CM) is well known as a rich source of autologous cytokines. Therefore, the use of MSC-CM may be a feasible approach to overcome these limitations.

Recent studies have shown the usefulness of MSC-CM in many kinds of diseases in vitro or in vivo. For example, Timmers et al. [22] found that human MSC-CM improves cardiac function following myocardial infarction. Chen et al. [23] reported that MSC-CM prevents radiation-induced liver injury by inhibiting inflammation and protecting sinusoidal endothelial cells. Tamari et al. [24] additionally suggested that the MSC-CM, containing growth factor derived from stem cells, is able to accelerate wound healing. Finally, Kim et al. [25] found that production of PGE2 by MSCs and subsequent production of IL-10 were required to reduce the severity of colitis.

The data presented in this study suggest that MSC-CM is a potential therapeutic treatment for vascular calcification. To the best of our knowledge, this is the first demonstration that MSC-CM can prevent beta-glycerophosphate (β-GP)-induced vascular smooth muscle cell (VSMC) calcification via downregulated BMP2 expression and blockade of the BMP2 signaling cascade. We found that MSC-CM inhibited calcification and osteoblast differentiation markers in cultured VSMCs.

In this study, we established an in-vitro model of VSMC calcification using β-GP. The calcification and calcium content showed a dose-dependent increase in the presence of 10, 20, and 40 mM of β-GP, a finding that has been verified by many researchers [6, 7, 26]. We found that MSC-CM dramatically inhibited β-GP-induced VSMC calcification and calcium deposition as determined by calcium amounts and Alizarin Red staining. To further investigate the inhibitory effect of MSC-CM on VSMC calcification, we detected alkaline phosphatase (ALP, an important enzyme in early osteogenesis) activity and specific osteogenic markers which were associated with the transition of the bone formation phenotype (i.e., Runt-related transcription factor 2 (Runx2), msh homeobox 2 (Msx2), and osteocalcin (OC)). Accumulating studies have demonstrated that vascular calcification is associated with the upregulation of these makers [27–30]. The results demonstrate that MSC-CM abolished β-GP-induced ALP activity and expression of osteogenic markers in VSMCs. These findings indicate that MSC-CM may be considered as a therapeutic agent to prevent or treat vascular calcification.

Some different signaling pathways work in vascular calcification, such as the BMP2–Smad1/5/8, NF-κB–RANKL, Wnt–β-catenin, endothelin-1–endothelin receptor, and AMPK–mTOR pathways. In this article, we demonstrated that MSC-CM suppression of calcification may be mediated by expression of BMP2 and the BMP2 receptor–Smad1/5/8 signaling pathway. Bone morphogenetic proteins (BMPs) are a superfamily of transforming growth factor beta (TGF-β) and secretory growth factor, which are reported to have osteogenic actions and play important roles in bone formation. BMP-2 is an important molecule in the regulation of bone formation as well as vascular calcification. Previous studies have demonstrated that BMP-2 plays a crucial role in osteoblast differentiation in the development of atherosclerosis and calcific vascular lesions [31, 32]. In in-vivo models of arterial calcification, BMP-2 gene expression is upregulated in the aortic adventitia and atherosclerotic plaques [33]. In addition, BMP2 accelerated atherosclerotic intimal calcification in BMP2 transgenic/apoE knockout mice [34]. Blockage of BMP-2 action by the antagonist, Noggin, inhibits osteoblast differentiation and bone formation in vivo and in vitro [35]. BMP signaling has consequently been addressed as a therapeutic target in vascular diseases.

In this study, we assessed the inhibitory potential of MSC-CM regarding osteogenesis. The results demonstrated that VSMCs, when stimulated by 10 mM β-GP, resulted in increased BMP-2 expression. Conversely, this effect was inhibited by treatment with MSC-CM, suggesting that MSC-CM suppression of calcification is mediated by inhibiting the expression of BMP2.

Gene responses to BMPs are mediated by Smad transcription factors after being phosphorylated by the BMP receptor complex [36]. Upon binding of BMP2, the type-II receptor (BMPR-II) transphosphorylates the type-I receptor (BMPR-I) and the phosphorylated BMPR-I, in turn, phosphorylates a set of intracellular substrate signaling proteins collectively known as receptor-regulated Smads (R-Smads), Smad1, Smad5, and Smad8 [36–39]. Next, phosphorylated Smad1/5/8 dissociates from BMPR-I, forms a heteromeric complex with a common-partner Smad (Co-Smad), Smad4, and translocates from the cytoplasm into the nucleus where they regulate transcription of various target genes, including Runx2 and Msx2, to increase various kinds of osteogenic differentiation indicators [40, 41]. Several studies support that the BMP2–Smad1/5/8 signaling pathway has been involved in bone remodeling processes including osteoblast differentiation and osteoclastogenesis [42, 43]. Considering that BMP2 is closely associated with the Smad pathway, we hypothesized that the inhibitory effect of MSC-CM on VSMC calcification is mediated by Smad1/5/8 signaling. Therefore, we investigated whether the MSC-CM inhibition of calcification was related to the phospho-Smad1/5/8 signaling pathway.

In this study, when VSMCs were stimulated by β-GP, mRNA expression of BMPR-I and BMPR-II increased, although the increase was suppressed by MSC-CM. Western blot analysis further showed that phosphorylated Smad1/5/8 was markedly upregulated when induced by β-GP, and downregulated when treated with MSC-CM. Next we tested the expression of phosphorylated Smad1/5/8 in the nucleus and cytoplasm separately. Western blot analysis showed that phosphorylated Smad1/5/8 in control VSMCs was low in nuclear regions and higher in the cytoplasm. When VSMCs were stimulated by β-GP, phosphorylated Smad1/5/8 mainly became distributed in the nucleus, not in the cytoplasm. These results suggested that β-GP not only promoted the expression of phosphorylated Smad1/5/8, but also accelerated its translocation from the cytoplasm to the nucleus. Treatment with MSC-CM, on the other hand, reduced the nuclear presence of phospho-Smad1/5/8 and increased its presence in the cytoplasm. Together, the results suggest that MSC-CM inhibited the expression of phosphorylated Smad1/5/8 and its transfer from the cytoplasm to the nucleus, where it plays a role in osteoblast differentiation.

To further investigate the role of BMP2 and the inhibitory effect of MSC-CM on VSMC calcification, we performed an experiment by which VSMCs were treated with MSC-CM in the presence of human recombinant BMP-2 (rhBMP2). Previous research has shown that BMP-2 enhances elevated phosphate-induced calcification, but does not induce calcification under normal phosphate conditions [44]. Our research also showed the same results: rhBMP2 strengthened β-GP-induced calcification and, in addition, both rhBMP2 in DMEM and that in MSC-CM did not cause calcification without β-GP (results not shown). When we added rhBMP2 in MSC-CM with β-GP, it overcame the suppression of MSC-CM on β-GP-induced VSMC calcification. We observed that rhBMP2 improved the calcium deposition and calcium content in the VSMCs which was inhibited by MSC-CM. rhBMP2 also upregulated several genes involved in osteoblastic transformation of VSMCs, which had been previously reduced by MSC-CM. rhBMP2 also significantly increased Runx2 and phospho-Smad1/5/8 protein expression inhibited by MSC-CM. These results demonstrated that MSC-CM inhibited VSMC calcification by suppressing BMP2.

Next, we demonstrated that BMP2 is implicated in the regulation of calcification, using the BMP2 antagonist Noggin to show the relevance of BMP2 in VSMC calcification. Noggin was identified as a BMP4 antagonist as well as other BMPs including BMP2, BMP7, and BMP14. In this study, the results show that Noggin inhibited β-GP-induced VSMC calcification, calcium content, and the associated marker genes, such as BMP2, Msx2, Runx2, and osteocalcin, suggesting that BMP2 and its downstream signaling are involved in the process of VSMC calcification.

Although our study shows that MSC-CM inhibition of calcification may be mediated via the regulation of BMP2 expression, we did not know which active component secreted by MSC-CM is responsible for calcification inhibition. Many research studies showed that MSC-CM contains various cytokines, such as insulin-like growth factor (IGF)-1, vascular endothelial growth factor (VEGF), and interleukin (IL)-10, which may play crucial roles in treatment of multiple diseases. Our results also showed that MSC-CM contained conspicuous high levels of IGF (74.4 ± 1.2 vs 0.09 ± 0.01 pg/ml), VEGF (813.1 ± 19.5 vs 0.0 ± 0.0 pg/ml), and IL-10 (98.9 ± 2.7 vs 6.9 ± 2.3 pg/ml) compared with the NC group, which may work in inhibiting VSMC calcification. However, the elucidation of the exact molecular mechanism responsible for cytoprotection can only be defined by another complex and long-term undertaking beyond the scope of this article.

## Conclusion

This study is the first to demonstrate that MSC-CM exerts significant protective effects on VSMC calcification. MSC-CM elicits this protection through reduction of BMP2-dependent Smad1/5/8 signaling, which plays a pivotal role in vascular calcification. This study could potentially extend the repertoire of stem cell-based therapy, as the data suggest that secretions of stem cells may offer a viable alternative to circumvent the complications associated with clinical cell-based therapy such as immunocompatibility, tumorigenicity, ethics, costs, and waiting time for ex-vivo expansion. In order to investigate that application further, however, the components secreted by MSC-CM which are responsible for cytoprotection must be studied further. These findings are the first to implicate MSC-CM as a contributor of a protective effect against calcification in vitro and identify a novel potential therapeutic for vascular calcification and its associated diseases.

## Abbreviations

ALP: Alkaline phosphatase
BMP-2: Bone morphogenetic protein-2
DMEM: Dulbecco’s Modified Eagle’s Medium
IGF-1: Insulin-like growth factor-1
IL-10: Interleukin-10
MSC: Mesenchymal stem cell
MSC-CM: Conditioned medium from bone marrow-derived mesenchymal stem cells
Msx2: Msh homeobox 2
OC: Osteocalcin
rhBMP2: Human recombinant BMP-2
Runx2: Runt-related transcription factor 2
VEGF: Vascular endothelial growth factor
VSMC: Vascular smooth muscle cell
β-GP: Beta-glycerophosphate
